# Dual-target VIM–PSA deep brain stimulation using octopolar electrodes: surgical technique and feasibility study

**DOI:** 10.3389/fneur.2026.1865673

**Published:** 2026-08-05

**Authors:** Lucía Triguero-Cueva, José Pablo Martínez-Barbero, Carlos Javier Madrid-Navarro, María José Pérez-Navarro, Adolfo Mínguez-Castellanos, Bartolomé Marín-Romero, Benjamín Iáñez-Velasco, Francisco Escamilla-Sevilla, Majed Jouma Katati

**Affiliations:** 1 Department of Neurology, Hospital Universitario Virgen de las Nieves, Granada, Spain; 2 IInstituto de Investigación Biosanitaria ibs. GRANADA, Granada, Spain; 3 Department of Neuroradiology, Hospital Universitario Virgen de las Nieves, Granada, Spain; 4 Neuropsychology, Hospital Universitario Virgen de las Nieves, Granada, Spain; 5 Department of Neurosurgery, Hospital Universitario Virgen de las Nieves, Granada, Spain; 6 Department of Surgery, Faculty of Medicine, Granada University, Granada, Spain

**Keywords:** deep brain stimulation, dentato-rubro-thalamic tract, double-target, essential tremor, octopolar electrodes, posterior subthalamic area, ventral intermediate nucleus

## Abstract

**Background/objectives:**

Deep brain stimulation (DBS) is an established surgical treatment for essential tremor (ET). Both the thalamic ventral intermediate nucleus (VIM) and the posterior subthalamic area (PSA) are effective tremor targets can be approached through a single trajectory. This technical feasibility report describes a single-trajectory dual-target VIM–PSA DBS surgical protocol using octopolar electrodes and evaluates stereotactic accuracy, anatomical contact distribution, and preliminary surgical safety.

**Methods:**

Eleven patients with medication-refractory ET underwent bilateral DBS implantation within the framework of an ongoing randomized, double-blind, crossover clinical trial comparing VIM-DBS and PSA-DBS. Preoperative planning was performed using Brainlab Elements, and postoperative reconstruction was performed using Guide™ XT after fusion of preoperative MRI with one-month postoperative CT. Clinical and electrical data (efficacy outcomes, stimulation thresholds, side-effect thresholds, active contact selection, energy consumption, habituation, patient-reported outcomes, and programming burden) were not assessed in this report.

**Results:**

A total of 22 octopolar DBS leads were implanted. Postoperative reconstruction confirmed anatomical contact distribution across the reconstructed VIM model and the PSA/cZI target region. Mean planned-to-implanted deviation across contacts was 1.28 mm for the left electrodes and 1.50 mm for the right electrodes. One patient developed transient symptomatic perielectrode edema; no intracerebral hemorrhage or any other major surgery-related adverse event was observed during early postoperative follow-up.

**Conclusion:**

Single-trajectory dual-target VIM–PSA DBS using octopolar electrodes was successfully performed with acceptable stereotactic accuracy and without major surgery-related adverse events in this small cohort. The approach may increase anatomical coverage and postoperative programming options. Clinical data remain to be determined.

## 1 Introduction

Medical treatment of essential tremor (ET) is often unsatisfactory or limited by side effects in many patients (1). Functional surgery of the thalamic ventral intermediate nucleus (VIM), including lesional therapies and deep brain stimulation (DBS), can be an alternative for many of these cases (2). Lesional procedures have become relevant again in recent years thanks to the novel magnetic resonance-guided focused ultrasound (MRgFUS) (2–4). Nevertheless VIM-DBS is still acknowledged as the first line recognised long-term treatment option. This is mainly due to its risk–benefit balance, and its potential for adjustment and reversion (2, 5, 6). However, side effects and loss of long term effect due to tolerance can limit the net benefits of the procedure (7–9).

In recent years, the posterior subthalamic area (PSA) has gained relevance as a potential DBS target (10, 11), with growing evidence from randomized clinical trials pointing to its efficacy and efficiency in comparison with VIM-DBS (12–14). PSA is a virtual area with no clearly distinguishable features on magnetic resonance imaging (MRI) and its stereotactic coordinates provided in the literature are not well defined (10, 15). It includes the tightly packed fibers of the dentato-rubro-thalamic tract (DRTT), thought to have a crucial role in the pathophysiology of tremor (15–18). Indeed, some studies have shown that the efficacy of DBS in ET is influenced by the distance to the DRTT (19). Details of the common DBS targets for ET are listed in Supplementary Table 1.

Therefore, the question of which target would be best for DBS in ET patients remains open. Nonetheless, as PSA is located ventromedial to VIM both these areas can be targeted with a single trajectory from lateral anterosuperior to medial posteroinferior. There are several reports of dual-target stimulation along the VIM–PSA trajectory in the literature, emphasizing how it facilitates stimulation of either target in the same patient. However, the use of tetrapolar electrodes in these reports may have an impact on the results, limiting the anatomical coverage for stimulation (12–14, 17, 20). More recently, preliminary long-term clinical data on bilateral VIM–cZI DBS using octopolar electrodes have further supported the potential utility of dual-target stimulation in complex tremor populations (21). Nevertheless, head-to-head comparisons between conventional single-target DBS and dual-target VIM–PSA approaches are still lacking.

We describe a surgical protocol for dual-target VIM–PSA DBS in ET using octopolar electrodes implanted along a single trajectory. The present manuscript reports technical feasibility, stereotactic accuracy, anatomical contact distribution, and preliminary surgical safety.

## 2 Methods

### 2.1 Study design and participants

This study is a prospective technical feasibility, stereotactic accuracy, and preliminary surgical safety analysis of a surgical protocol for dual-target VIM–PSA DBS in patients with ET. The protocol was implemented within the framework of a randomized, double-blind, crossover clinical trial evaluating PSA-DBS versus VIM-DBS in ET (ClinicalTrials.gov identifier: NCT07526155). The study was approved by the Provincial Ethics Committee of Granada (Spain), and all procedures were conducted in accordance with the Declaration of Helsinki. Written informed consent was obtained from all participants. The present manuscript reports the surgical technique and technical evaluation of the implantation approach; clinical outcomes from the registered trial will be reported separately.

Eligible participants were patients with disabling bilateral ET or ET-plus refractory to pharmacological treatment, who were evaluated by two expert neurologists and considered suitable candidates for bilateral DBS implantation. Eligibility criteria for the clinical trial, including diagnostic criteria, medication refractoriness, cognitive and psychiatric exclusion criteria, surgical contraindications, and neuroimaging or anatomical exclusion criteria relevant to stereotactic targeting, are summarized in Supplementary Table 2. Additional details regarding the clinical trial design and participant characteristics were previously reported (22). All included patients underwent bilateral octopolar electrodes implantation (Vercise™ Standard Lead DB2201-45-DC, Boston Scientific, USA) in a single trajectory including the VIM (proximal contacts) and PSA (distal contacts).

### 2.2 Image acquisition and pre-processing/surgical planning strategy

Prior to surgery, patients underwent a 3-T MRI scanner (Ingenia-Cx, Philips, Erlingen), using a 32-channel head-coil. The acquisition protocol (Supplementary Table 3) included T1-3D SPGR, T2-Brain-View-3D, 32-direction diffusion tensor imaging (DTI; *b* = 0 and *b* = 800), and post-contrast T1-3D SPGR (gadoteric acid 0.5 mmol/mL, at a dose of 0.2 mL/kg body weight) (23–26).

The DRTT was delineated using the fiber-tracking software package on the Brainlab platform (BrainlabAG, Munich, Germany), using a deterministic fiber-tracking (dFT) algorithm, with the red nucleus (RN), VIM and dentate nucleus as regions of interest. Uncrossed DRTT (U-DRTT; all three ipsilateral structures) and crossed DRTT (C-DRTT; ipsilateral RN and VIM with contralateral dentate nucleus) were reconstructed bilaterally. DRTT tractography was not used as the primary method for target localization. Instead, it was used as a complementary tool to assess the anatomical relationship between the planned trajectory and the tremor-related network during surgical planning, and later to interpret the postoperative relationship between electrode contacts and the reconstructed tract.

Same-day stereotactic planning was performed prior to surgery. Ad-hoc imaging was acquired with an intraoperative O-arm image acquisition system (iCT) after stereotactic frame fixation (Integra Cossman-Robert-Wells, CRW ®) under local anesthesia. Final planning images were obtained by fusing the newly acquired stereotactic iCT with the preoperative MRI. Intraoperative O-arm CT/iCT was also repeated after definitive electrode implantation to verify the final electrode location before completion of the surgical procedure. Supplementary Figure 1 shows the intraoperative O-arm imaging procedure.

### 2.3 Target planning

Target and trajectory planning were performed on the surgical date using the Brainlab Elements® planning station (Brainlab Elements® - Rigid Image Fusion - BrainlabAG, Munich, Germany), based on volumetric MRI image fusion (T1WI, Gd-T1WI and T2WI).

**Figure 1:**
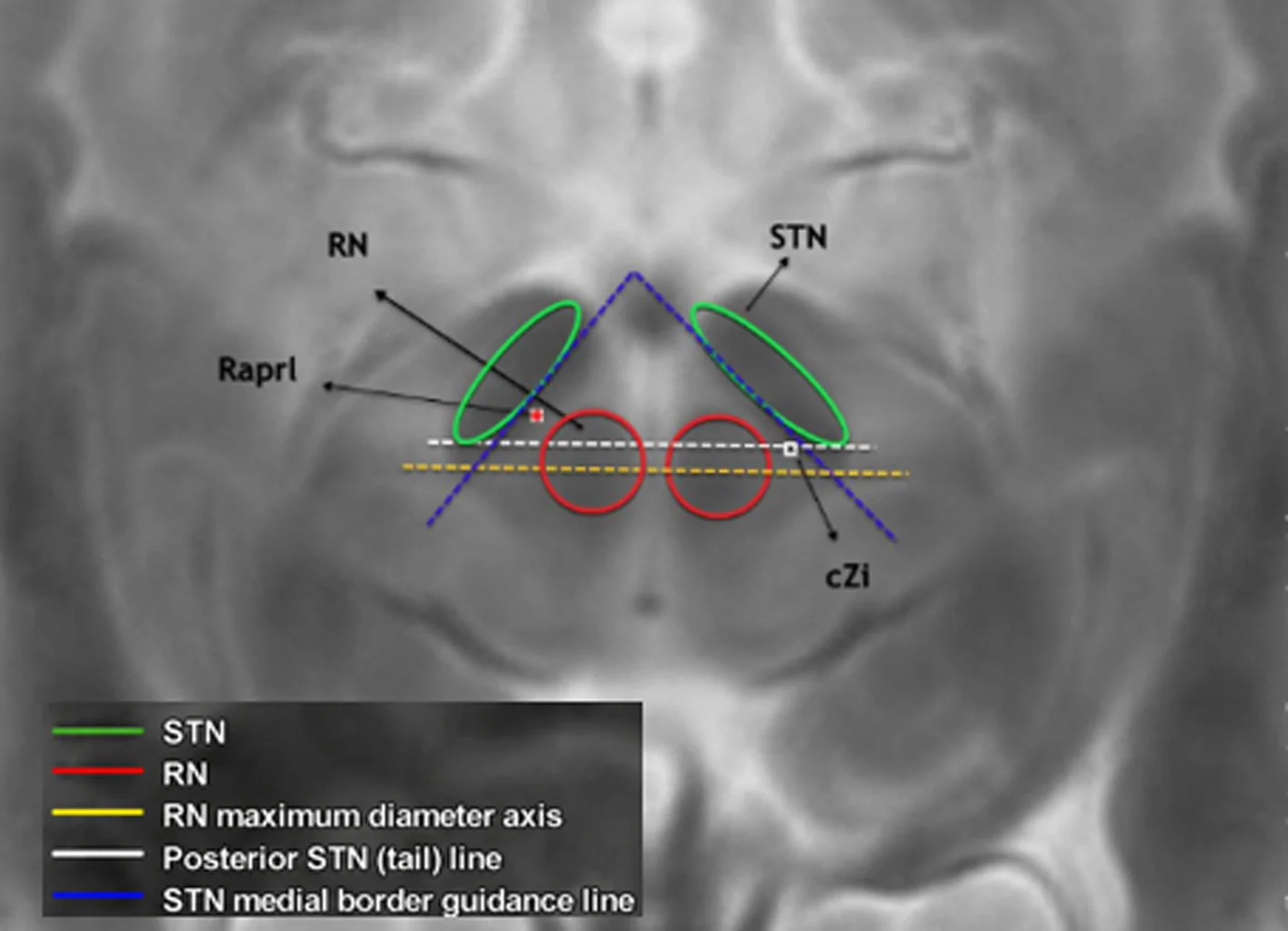
T2-weighted MRI sequence and target planning for cZI localization. RN, Red nucleus; STN, Subthalamic nucleus; Raprl, Prelemniscal radiation; cZI, Caudal zona incerta. The yellow reference line follows the bilateral RN reference axis used during planning; the blue line follows the medial border of the STN; and the white line is drawn parallel to the yellow line through the posterior tail of the STN. The cZI target is located approximately 1 mm medial to the intersection between the blue and white lines (white dot). The figure illustrates the planning landmarks on a representative slice; final target localization was performed using multiplanar stereotactic planning.

The PSA was defined as the primary target. Within the PSA, the caudal zona incerta (cZI) was selected as the preferred stimulation region and was used as the anatomical surrogate for PSA in target planning and postoperative reconstruction. The cZI target was localized on T2-weighted MRI using anatomical landmarks based on the red nucleus, subthalamic nucleus, and posterior tail of the subthalamic nucleus, as illustrated in Figure 1 and described in its legend. The prelemniscal radiation (Raprl) was considered an adjacent component of the PSA region that could inform trajectory adjustment in selected anatomical situations, but it was not used as the primary reconstruction surrogate. Regarding the VIM, the classical stereotactic coordinates were used as a reference: 12–14 mm lateral, 4–6 mm posterior, and 0–1 mm inferior to the midcommissural point (20, 27).

Finally, when trajectory adjustments were required in the planning, they were achieved by modifying coronal and sagittal angles to align the trajectory with both intended targets. The relationship of the planned trajectory with the individually reconstructed DRTT was reviewed as a complementary anatomical verification step, although DRTT tractography was not used as the primary determinant of target localization (Supplementary Figure 2). Subtle adjustments may also be necessary to avoid certain functional areas or vascular structures. Planned VIM and PSA/cZI target coordinates relative to the midcommissural point in AC-PC space were recorded and are summarized in Supplementary Table 4.

As a general point of practice, the entry point for a dual VIM/PSA aligned trajectory is more posterior and lateral than for single VIM targeting.

### 2.4 Surgical procedure

Electrode implantation was performed under local anesthesia on awake patients, starting on the left hemisphere as per protocol.

Once the VIM–PSA double-target planning was completed, exquisite accuracy of the location of the entry burr hole (a standard 14 mm diameter) and the entry point in the cerebral cortex was paramount, as this step is a critical requirement for accurate target alignment in the planned trajectory. Furthermore, surgical technique aims to minimize pneumocephalus and CSF loss by including measures such as discrete head elevation, an “arachnoid-piamater welding” incision (20) and continuous saline irrigation throughout the procedure.

On the surgical date, the stereotactic frame was fixed to the patient’s head under local anesthesia prior to preoperative iCT. Then, the patient was placed in a semi-supine position with the head elevated about 15 degrees at the center of the O arm.

To ensure optimal accuracy, the stereotactic guide was placed, used and removed at each of the following steps during the surgical procedure: 1. to locate the entry point on the scalp, where a curved incision is performed; 2. to locate the center point of the burr hole and 3. to center the entry point into the cortex. The dura was opened with a transverse incision, leaving the arachnoid intact (the arachnoid and dura are coagulated together and cut).

Intraoperative microelectrode recording (MER) (NeuroTrek system, AlphaOmega, Nof HaGalil, Israel) was used to refine localization along the planned VIM–PSA trajectory. Microregistration was usually started 10 mm above the theoretical target. VIM identification was supported by the presence of typical thalamic neuronal activity along the planned trajectory and, when present, tremor-related bursting activity. Progression toward the ventral part of the trajectory was characterized by a transition to a lower-activity region, interpreted as the neural silence zone corresponding to PSA/cZI. After electrophysiological mapping and after identifying the theoretical PSA-target, a test stimulation was performed by applying monopolar stimulation through the microelectrode to assess tremor suppression and stimulation-induced side effects. If the intraoperative electrophysiological and clinical findings were favourable, the definitive octopolar electrode was implanted and macrostimulation was performed to assess the effect and side-effect profile of both proximal and distal electrode contacts. The final electrode position was accepted when the overall electrophysiological, anatomical, and clinical intraoperative findings were considered favourable. Final location of the electrode was determined by a subsequent iCT scan. The same procedure was repeated for contralateral electrode implantation. After removal of the head frame, an abdominal subcutaneous impulse generator is implanted under general anesthesia (Boston Vercise™ PC DB1416).

### 2.5 Data analysis

To reduce the influence of postoperative pneumocephalus and early postoperative brain shift on image fusion and electrode localization, postoperative cranial CT was performed 1 month after surgery. We used a 64-detector General Electric Revo/Evo CT scanner with a dedicated head holder. Image reconstructions were obtained with 5-mm standard, 0.625-mm standard, and 0.625-mm bone-plus kernels, without inter-slice gap. The acquisition pitch was 0.531:1, with 240 mA and variable kV according to automatic dose modulation. Postoperative reconstruction was performed using Guide™ XT software (Boston Scientific, Marlborough, MA) after fusion of the preoperative MRI with the one-month postoperative cranial CT. Electrode location was reconstructed in three dimensions and assessed in relation to the reconstructed VIM model, the PSA/cZI target region, the reconstructed DRTT, and adjacent anatomical structures. Because VIM and PSA/cZI are not directly visible on conventional MRI, postoperative VIM and PSA/cZI localization was based on a software-generated anatomical model registered to the patient’s stereotactic space after image fusion. Therefore, VIM and PSA/cZI contacts coverage in this study refers to contact localization relative to the reconstructed VIM and PSA/cZi model. Contact-structure relationships were interpreted as anatomical proximity or overlap within the reconstruction environment, not as evidence of functional stimulation or clinical effect.

Analysis of the anatomical deviation between planned and implanted electrode locations was performed contact by contact using Guide™ XT software. Planned and implanted electrodes were reconstructed in the same stereotactic space. The geometric center of each planned and implanted contact was visually identified using axial, sagittal, and coronal views, and measurements were refined in oblique planes aligned with the electrode axis, including para-coronal and para-sagittal views when required. For each contact, the planned-to-implanted deviation was calculated as the distance between the corresponding planned and implanted contact centers on the plane perpendicular to the electrode trajectory. Measurements were performed by a single evaluator. Numerical data are presented as mean ± standard deviation.

For the purpose of the reconstruction analysis, a contact was considered “anatomically related” to a reconstructed structure when any portion of the reconstructed contact touched or overlapped the boundary of the corresponding structure within the Guide XT reconstruction environment. Thus, contacts were not required to be fully surrounded by the target, and no predefined volumetric threshold was applied. This analysis was intended to describe anatomical contact coverage along the VIM–PSA/cZI trajectory and the availability of potentially programmable contacts touching or overlapping each target region. This intentionally permissive criterion may overestimate the number of clinically viable contacts, since a contact only marginally touching the target boundary may be predominantly surrounded by adjacent structures potentially associated with stimulation-induced side effects. Therefore, contacts classified as anatomically related should be interpreted as potentially available contacts with at least minimal target contact, rather than as definitively effective or safe stimulation contacts or evidence of VTA overlap.

## 3 Results

Between 2019 and 2024, a total of 22 DBS leads were placed in 11 ET patients. Baseline demographic and clinical characteristics relevant to surgical feasibility and generalizability are summarized in Supplementary Table 5. All patients underwent bilateral electrode implantation. Regarding surgical complications during the perioperative and early postoperative follow-up period, one patient developed transient symptomatic perielectrode edema. No intracerebral haemorrhages or other surgery-related adverse events was observed in this cohort.

### 3.1 Final electrode location in relation to planned trajectories and VIM–PSA targets

**Figure 2:**
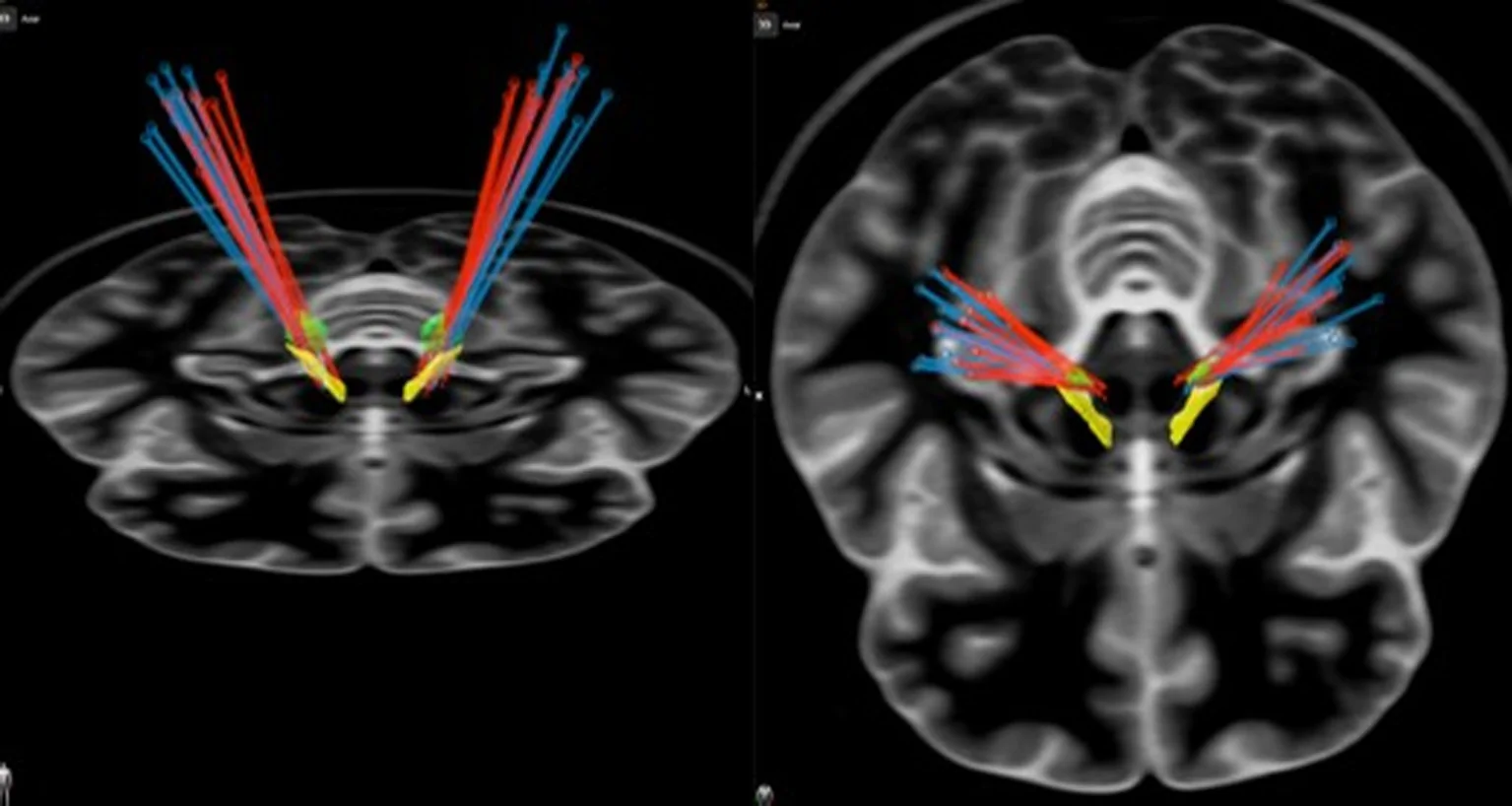
Representation of the 22 electrodes in their final position (red lines) compared to the planned (blue lines), and their relation to VIM (green) and PSA/cZI (yellow), after intraoperative corrections in some cases based on intraoperative microelectrode recording (MER) and clinical evaluation. This figure provides a qualitative visualization; directional deviations should not be inferred from this projection alone.

Figure 2 (and Supplementary Figure 3) show the final position of the electrodes in relation to the planned trajectories and VIM–PSA targets after reconstruction with the Guide™ XT software based on postoperative CT and preoperative MR images.

All implanted electrodes had at least one contact anatomically related to the reconstructed VIM model and at least one contact related to the PSA/cZI target region; therefore, all operated patients entered the subsequent clinical trial.

Figure 2 provides a qualitative visualization of planned and final electrode trajectories after possible intraoperative corrections based on MER and clinical evaluation. Directional deviations should not be inferred from this projection alone. Quantitative accuracy was assessed using contact-by-contact planned-to-implanted deviation analysis and is shown in Table 1 as mean ± standard deviation. Mean planned-to-implanted deviation across contacts was 1.28 mm for left electrodes and 1.50 mm for right electrodes. In an exploratory paired contact-level analysis comparing mean deviations from contact 8 to contact 1, right-sided deviations were slightly higher than left-sided deviations, with a mean paired difference of 0.21 mm and a median paired difference of 0.21 mm (Wilcoxon signed-rank test, *p* = 0.008). However, this analysis was based on contact-level mean values and should not be interpreted as a patient-level or electrode-level comparison. Overall, planned-to-implanted deviation remained relatively stable along the electrode length, with similar values across proximal and distal contacts. As an additional exploratory accuracy analysis, 40 of 176 contacts (22.7%) were misplaced by > 2 mm in this study cohort, included 24/88 contacts (27.3%) in the left hemisphere and 16/88 contacts (18.2%) in the right hemisphere.

**Table 1:** Deviation between planned and final location of each of the implanted contacts.

Contact	Average of the distances planned-implanted (mm ± sd)	
	Left electrodes	Right electrodes
Contact 8	1.35 ± 1.71	1.55 ± 1.97
Contact 7	1.30 ± 1.70	1.55 ± 1.96
Contact 6	1.26 ± 1.72	1.53 ± 1.97
Contact 5	1.25 ± 1.70	1.51 ± 1.94
Contact 4	1.26 ± 1.67	1.49 ± 1.91
Contact 3	1.28 ± 1.68	1.47 ± 1.91
Contact 2	1.30 ± 1.67	1.45 ± 1.89
Contact 1	1.28 ± 1.67	1.45 ± 1.86

Contact 8 is the most dorsal and contact 1 is the most ventral.

**Figure 3:**
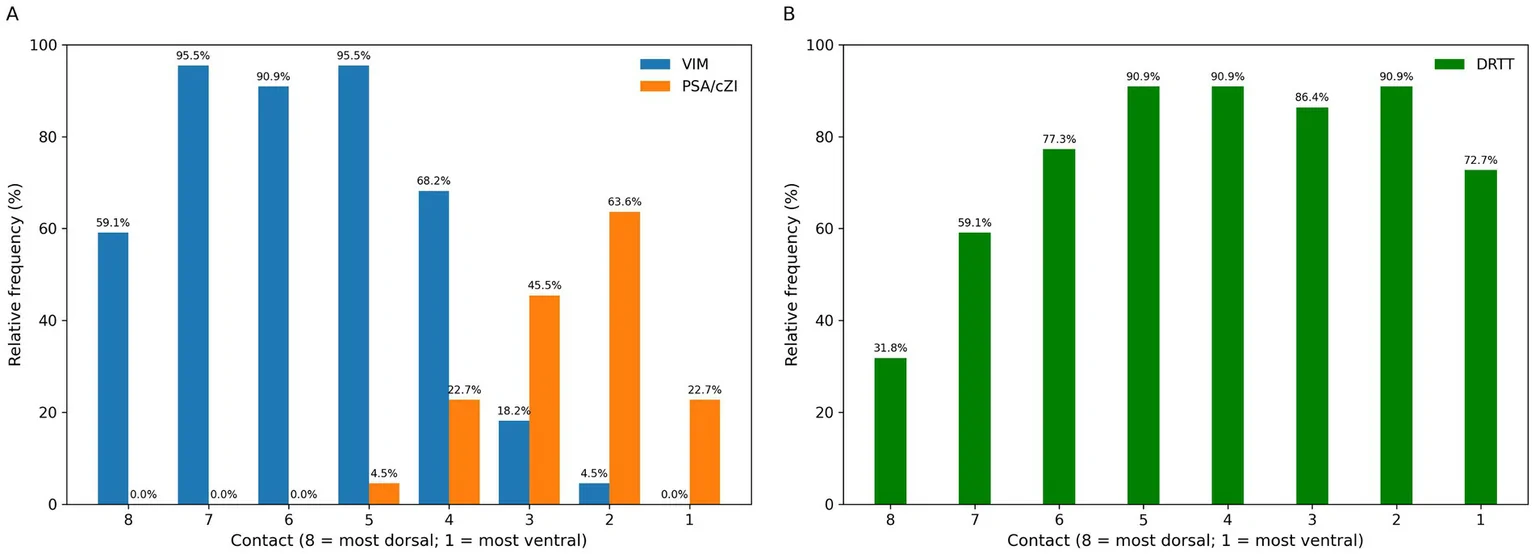
Contact distribution according to reconstructed anatomical and tractographic structures. Contact 8 is the most dorsal contact and contact 1 is the most ventral contact. **(A)** Shows the relative frequency of electrode contacts anatomically related to the reconstructed VIM model and the PSA/cZI target region. **(B)** Shows the relative frequency of electrode contacts anatomically related to the reconstructed DRTT tractography. Values represent relative frequencies (%), calculated using 22 contacts per contact level as the denominator. “Anatomically related” indicates that any portion of the reconstructed contact touched or overlapped the boundary of the corresponding structure. These anatomical contact–structure relationships should not be interpreted as functional stimulation data.

As previously mentioned, reconstruction analysis was performed using cZi as the anatomical surrogate for the PSA target region. The relationship between electrode contacts and the reconstructed VIM model, the PSA/cZI target region, and DRTT tractography was quantified separately. The analysis showed that out of the 88 right hemisphere electrode contacts, 48 were anatomically related to the reconstructed VIM, 15 to the PSA/cZI and 74 showed anatomical relationship with the reconstructed DRTT. Out of the 88 left hemisphere contacts, 47 were anatomically related to the reconstructed VIM, 18 to the PSA/cZI and 58 showed anatomical relationship with the reconstructed DRTT. These findings should be interpreted as anatomical relationships with reconstructed anatomical or tractographic structures, not as evidence of functional stimulation. The PSA target was mainly related to ventral contacts 1–3 (80% of cases), whereas the VIM target was mainly related to dorsal contacts 4–8 (90% of cases). All electrodes had some of their contacts anatomically related to the reconstructed DRTT; the most dorsal contacts (contacts 7 and 8) showed the least anatomical relationship (Figure 3).

Determination of electrode placement relative to reconstructed anatomical structures may inform strategic programming of brain stimulation within the planned clinical trial. Clinical efficacy outcomes, stimulation thresholds, side-effect thresholds, active contact selection, energy consumption, habituation, patient-reported outcomes, and programming burden will be reported once the trial is completed.

## 4 Discussion

### 4.1 Aligning VIM and PSA

Single-trajectory dual alignment of VIM and PSA is feasible in selected cases and there are favourable reports on this dual-target surgical strategy (12, 14, 17, 20, 21, 27–29), including previous experience with 8-contact leads (21, 28, 29). These publications highlight the clinical interest of dual-target approaches and support the relevance of investigating broader anatomical coverage along the VIM–PSA trajectory. However, there is scarce literature that includes the detailed technical aspects (14, 17, 20, 27), which is in line with our strategy. The present manuscript is limited to the technical description, stereotactic accuracy, planned-coordinate reporting, anatomical contact distribution, and preliminary surgical safety of a single-trajectory VIM–PSA approach using octopolar electrodes within the framework of an ongoing randomized clinical trial.

The VIM–PSA dual-target surgical strategy may offer certain advantages. On one hand, it could allow comparison between targets, using the same patient as a control. On the other hand, may increase the range of anatomical regions available for postoperative stimulation and may provide additional programming option, which could help to improve the therapeutic range and the threshold for adverse effects. In addition, aligned trajectories are possibly in closer relationship with the DRTT, which seem to be an optimal target of stimulation in ET (19, 30). However, whether this translates into a wider therapeutic window, fewer stimulation-related adverse effects, reduced programming burden, or superior clinical outcomes remains to be determined.

Conversely, as a disadvantage, the predefined coverage of two target sites with a single trajectory might lead to a compromise for optimal targeting of VIM or PSA depending on the entry point, which needs to take into account the cortical gyration and vascularization along the electrode path.

In our study, VIM–PSA alignment was achieved in all implanted electrodes, according to the post-surgical reconstruction data.

### 4.2 Electrode selection

There is growing evidence on dual-target VIM–PSA with quadripolar electrodes, whereas reports on octopolar electrodes remain limited (21, 28, 29). Previous studies have suggested that four-contact electrodes may be insufficient to fully cover the VIM–PSA region, potentially limiting the range of stimulation options, particularly at more distal levels (20). In contrast, the use of an eight-contact electrode may provide broader anatomical coverage (15.5 mm vs. 7.5 or 10 mm for the four-contact electrodes). This broader spatial distribution may increase the number of contacts available for postoperative programming.

Based on these considerations, we decided to implant bilateral octopolar electrodes maximize anatomical coverage of the intended VIM–PSA trajectory. Postoperative reconstruction confirmed that all electrodes had contacts distributed across the VIM–PSA, with additional anatomical relationship to the reconstructed DRTT. In our small cohort, the use of octopolar electrodes was not associated with an increased rate of surgical complications compared with our experience with quadripolar electrodes. However, the present study did not include a direct comparison with quadripolar electrodes. Therefore, no conclusions can be drawn regarding the superiority of octopolar leads in terms of efficacy, efficiency or safety.

In this context, the segmented octopolar electrodes may further enhance the anatomical and programming potential of this approach by enabling directional stimulation across both targets. The increased number of available contacts may expand programming options but may also increase programming complexity. Image-guided and algorithm-assisted programming tools, such as Illumina 3D and related platforms, may help integrate patient-specific anatomy, lead reconstruction, target regions, and avoidance regions to guide initial programming strategies. Comparative studies are required to determine whether broader anatomical coverage and a greater number of available contacts translate into clinically meaningful benefits.

### 4.3 Accuracy of electrode implantation

Electrode implantation accuracy is closely related to clinical efficacy. Deviations of more than 2 mm from the planned coordinates have been reported by Papavassiliou et al. (31) to be associated with poor tremor control. It is therefore necessary to be rigorous at every step of the surgical process, to minimize deviation from the planned trajectories. In our study, mean planned-to-implanted deviation across contacts was below 2 mm on both sides (Table 1), although the limited dataset and measurement method should be considered when interpreting this result. Figure 2 provides a qualitative visualization of planned and final trajectories. Although final trajectories may appear slightly more medial in some cases, this was not formally assessed using directional vector analysis. This apparent trend may reflect intraoperative trajectory refinements based on MER findings, test stimulation, and clinical assessment, including avoidance of capsular side effects.

As an additional exploratory accuracy analysis, 22.7% of contacts exceeded a conservative >2-mm deviation threshold. This finding should be interpreted with caution. The 2-mm cutoff is useful as a general benchmark for DBS lead placement accuracy, but it should not be considered a strict threshold for clinical failure or lack of safety at the individual contact level. Moreover, planned-to-implanted deviation may not always represent unintended surgical error in MER-guided awake DBS procedures. In the present study, part of the deviation from the initial planned trajectory may reflect deliberate intraoperative refinements based on MER findings and micro- or macrostimulation responses, including attempts to optimize tremor suppression and avoid stimulation-induced side effects related to adjacent structures such as the internal capsule. Therefore, the clinical relevance of contacts exceeding this accuracy reference remains uncertain in this technical feasibility report and will need to be interpreted together with stimulation thresholds, side-effect thresholds, active contact selection, and clinical outcomes.

In the surgical phase there are several strategies that can be implemented to improve electrode implantation accuracy. Firstly, unlike single-target DBS surgery, accuracy in determining the electrode trajectory for dual-target DBS and maintaining it throughout the surgical procedure are paramount. This is achieved by using stereotactic guidance to plan the burr hole and open the cerebral cortex. Secondly, to minimize CSF leakage and reduce pneumocephalus and spatial distortion, the procedure includes discrete head elevation and continuous saline irrigation throughout the intervention. The surgical arachnoid incision technique performed is similar to the one proposed by Hou et al. (20), referred to as ‘arachnoid-piamater welding’. Despite these measures, our results showed that mean planned-to-implanted deviation was slightly greater on the right side than on the left (although the difference was small). This is possibly due to ‘brain shift’ in the second trajectory, which in our protocol is always the right side. Thirdly, intraoperative MER and macrostimulation under local anesthesia help guide electrode placement, enabling precise targeting and real-time assessment of clinical response and stimulation-induced side effects. Finally, intraoperative O-arm/iCT was used to verify trajectory and placement against preoperative plans; prior studies have supported the accuracy of this method compared with postoperative MRI (32).

In the pre-surgical phase, based on our experience, we propose two basic principles for the subtle adjustment of the trajectory to both aligned targets. Firstly, we usually target the cZI (most lateral area of the PSA) for a medial trajectory and smaller coronal angle, reducing risks to the insula, internal capsule and language areas. We target the Raprl (medial component of the PSA) for a lateral trajectory, in patients with ventricular enlargement. Both cZI and Raprl stimulation are equally effective for tremor control (33). In addition, if the sagittal angle is too steep, the VIM target can be adjusted 1 mm anteriorly to avoid motor or functional areas.

### 4.4 DRTT DTI-tractography

The use of tractography for DBS is a promising technique to optimize surgical outcome. Some studies have advocated direct target localization using DTI tractography (30). However, variability in stereotactic software, ROIs and image acquisition parameters between different surgical teams may explain the possible variations in DRTT representations (23, 34–36). In our study, DRTT tractography was used as a complementary anatomical tool rather than as the primary method for target definitions. DRTT reconstruction was used to assess the relationship of the planned trajectory and final electrode location with the tremor-related network.

In our case, and unlike other authors (14, 17, 27), we acquired individual diffusion images using DTI, allowing us to generate patient-specific DRTT reconstructions. We used a deterministic method to generate DRTT bilaterally and performed both the U-DRTT and the C-DRTT reconstructions; however, it should be noted that deterministic DTI tractography has several limitations (including dependence on image acquisition parameters, ROI selection, software algorithms, tracking thresholds, crossing fibers, and operator-dependent decisions).

It would be interesting to conduct further studies on the efficacy of target determination using DRTT versus stereotactical target coordinates and structural MRI anatomy.

### 4.5 Surgical complications

In this small feasibility cohort, one patient developed transient symptomatic perielectrode edema and no others major surgery-related adverse event was observed during the perioperative and early postoperative follow-up period. This percentage of surgical complications is lower than that reported in the literature (12, 13, 17). However, the limited sample size precludes robust conclusions regarding the safety profile of this approach. Larger cohorts and longer follow-up are required to determine the true incidence of surgery-related complications.

### 4.6 Post-operative imaging processing

In line with the literature, postoperative CT is performed 1 month after surgery to minimize pneumocephalus artefact and allow more accurate fusion with preoperative planning MRI (20). Some groups use software-based correction, although we consider this method less reliable (14).

### 4.7 Limitations

The present study has several limitations. First, the number of patients included in this study is limited, which restricts the generalizability of the findings and limits the strength of any conclusions. The absence of major surgery-related adverse events in this cohort should therefore be interpreted as preliminary safety information rather than evidence of a definitive safety profile. Second, this study was designed to assess surgical feasibility, stereotactic accuracy, and preliminary safety, but it did not evaluate clinical or electrical data (tremor severity outcomes, stimulation thresholds, side-effect thresholds, active contact selection, energy consumption, habituation, patient-reported outcomes, programming burden, or long-term clinical benefit); these will be determined once the ongoing trial has been completed. Therefore, no conclusions can be drawn regarding this aspects. Third, VIM localization in postoperative reconstruction was based on a software-generated anatomical model registered to stereotactic space rather than direct visualization of the VIM on conventional MRI. Fourth, cZI was used as the preferred PSA stimulation region and as the anatomical surrogate for PSA in postoperative reconstruction; therefore, contact localization described in relation to PSA should be interpreted as contact localization relative to the reconstructed cZI model rather than direct segmentation of the entire PSA region. Fifth, DRTT tractography was analysed separately as a tremor network-related structure and was not considered interchangeable with PSA/cZI. Anatomical proximity or overlap between electrode contacts and the reconstructed DRTT should be interpreted cautiously and should not be considered equivalent to functional stimulation of the tract. Sixth, the planned-to-implanted deviation analysis was based on visual identification of the geometric center of each contact and was performed by a single evaluator. Inter-rater reliability was not assessed and fusion error was not quantitatively estimated. Seventh, the reconstruction analysis was based on a qualitative contact–structure criterion, whereby any contact touching or overlapping a reconstructed structure was considered anatomically related to it. No predefined volumetric threshold, VTA-based analysis, or independent rater assessment was performed. Therefore, these anatomical relationships should be interpreted as contact coverage data rather than evidence of functional target activation. This permissive criterion may overestimate the number of clinically viable contacts, since marginal overlap with a target does not ensure that stimulation would predominantly engage that structure or avoid adjacent regions potentially associated with stimulation-induced side effects. Accordingly, the reconstruction analysis should be interpreted as anatomical contact coverage rather than as a direct estimate of clinically effective or safe stimulation contacts. Finally, surgical strategy included techniques of arachnoid incision and burr hole sealing aimed at reducing pneumocephalus and CSF leakage. However, evidence supporting the effectiveness of these measures remains limited, and further comparative studies are required to validate their impact.

## 5 Conclusion

This feasibility study suggests that single-trajectory dual-target VIM–PSA DBS using octopolar electrodes can be performed with acceptable stereotactic accuracy and without major surgery-related adverse events in this small cohort. The approach suggests anatomical contact coverage across VIM and PSA/cZI regions and may increase postoperative programming options. Clinical efficacy, therapeutic window, stimulation-related adverse effects, programming burden, and long-term outcomes remain to be determined.

## Data Availability

De identified data may be made available from the corresponding author upon reasonable request and subject to applicable ethical and institutional restrictions.
