# Supplementary material for: Dual-target VIM–PSA deep brain stimulation using octopolar electrodes: surgical technique and feasibility study
Source: Front Neurol. 2026 Aug 5;17:1865673. doi: 10.3389/fneur.2026.1865673 (PMC13488162; doi:10.3389/fneur.2026.1865673)
Supplement: Supplementary file 1 [file Data_Sheet_1.PDF]

Supplementary Material

1 Supplementary Figures and Tables

1.1 Supplementary Tables

Supplementary Table 1. DBS targets for ET.

	Location or Component	Standard stereotactic coordinates (relative to the midcommissural point)
VIM [17, 20]	Thalamus	Lateral 12-14 mm<br>Posterior 4-6 mm<br>Inferior 0-1 mm
PSA [17, 18, 20]	Includes: zona incerta (Zi) and prelemniscal radiation (Raprl). Located: ventral to the thalamus, medial to the posterior tail of the subthalamic nucleus (STN) and lateral to the red nucleus (RN).	Lateral: 10-12 mm<br>Posterior: 6-8 mm<br>Inferior: 2-4 mm

Table modified from Hou et al [20].

Supplementary Table 2. Inclusion and exclusion criteria for the clinical trial.

**Inclusion criteria**	**Exclusion criteria**
Confirmed diagnosis of bilateral ET or ET-plus according to the Movement Disorders Society criteria.	Clinically relevant cognitive decline that could interfere with the study.
Refractoriness to medication, defined as at least two treatment attempts with at least two different medication groups, mainly propranolol and primidone, which were ineffective because of insufficient tremor control or adverse effects.	Clinically relevant active psychiatric disorder.
Subjects of both sexes, older than 18 years.	Contraindication for general surgery or bilateral DBS.

Sufficient competence to collaborate and comply with the study protocol.	Unsuitable electrode location according to neuroimaging.
Ability to provide written informed consent.	Participation in another interventional study.
	Cerebral atrophy, defined as third-ventricle width >10 mm, or other anatomical abnormalities that could interfere with optimal stereotactic localization.

Supplementary Table 3. Preoperative MRI parameters.

Sequences	Parameters
T1-3d SPGR	Voxel size: 1 x 1 x 1 mm<br>Matrix 256 x 256<br>TR: 7.9; TE: 3.5<br>Duration: 4 min 46 secs<br>Slices: 170
T2-Brain-View-3d	Voxel size: 0.6 x 0.6 x 0.6 mm<br>Matrix 288 x 288<br>TR: 2500 ms; TE: 29.49 ms<br>Duration: 4 min 32 secs<br>Slices: 238
DTI	Voxel size: 1.75 x 1.75 x 2 mm<br>Matrix 128 x 128<br>TR: 3879.97; TE: 87.77<br>Duration: 9 min 32 secs<br>Directions: 32<br>Slices: 63
Post-contrast T1-3d SPGR	Voxel size: 1 x 1 x 1 mm<br>Matrix 256 x 256<br>TR: 7.9; TE: 3.5<br>Duration: 4 min 46 secs<br>Slices: 170

Abbreviations: TR = repetition time; TE = echo time.

Supplementary Table 4. Planned VIM and PSA/cZI target coordinates relative to the midcommissural point.

Target	Hemisphere	Lateral, mm	Posterior, mm	Vertical, mm
**PSA/cZI**	Right	10.53 +/- 1.04 (9.42-12.74)	6.82 +/- 1.44 (5.20-9.89)	Inferior 4.29 +/- 1.54 (1.92-6.30)
**PSA/cZI**	Left	9.83 +/- 0.93 (8.14-11.24)	7.35 +/- 1.43 (6.03-10.40)	Inferior 5.07 +/- 0.86 (3.06-5.86)
**VIM**	Right	13.69 +/- 0.46 (13.15-14.77)	4.02 +/- 0.89 (2.53-5.59)	Inferior 0.98 +/- 0.24 (0.52-1.43)
**VIM**	Left	12.69 +/- 0.58 (11.49-13.65)	4.52 +/- 0.93 (3.38-6.48)	Inferior 0.76 +/- 0.43 (0.07-1.37)

Values are expressed as mean +/- standard deviation, with range in parentheses. Coordinates are reported relative to the midcommissural point in AC-PC space.

Supplementary Table 5. Baseline characteristics of the study cohort.

Characteristic	Study cohort (n = 11)
Age at surgery, years	63.18 +/- 7.59
Sex	
Female	6 (54.55%)
Male	5 (45.45%)
Disease duration, years	20.91 +/- 9.39
Right-handedness	11 (100%)
Family history of tremor	8 (72.73%)
Number of previous drugs	3.72 +/- 1.85
Baseline FTM-TRS total score	60.91 +/- 15.97

Values are expressed as mean +/- standard deviation or n (%). FTM-TRS = Fahn-Tolosa-Marin Tremor Rating Scale.

1.2 Supplementary Figures

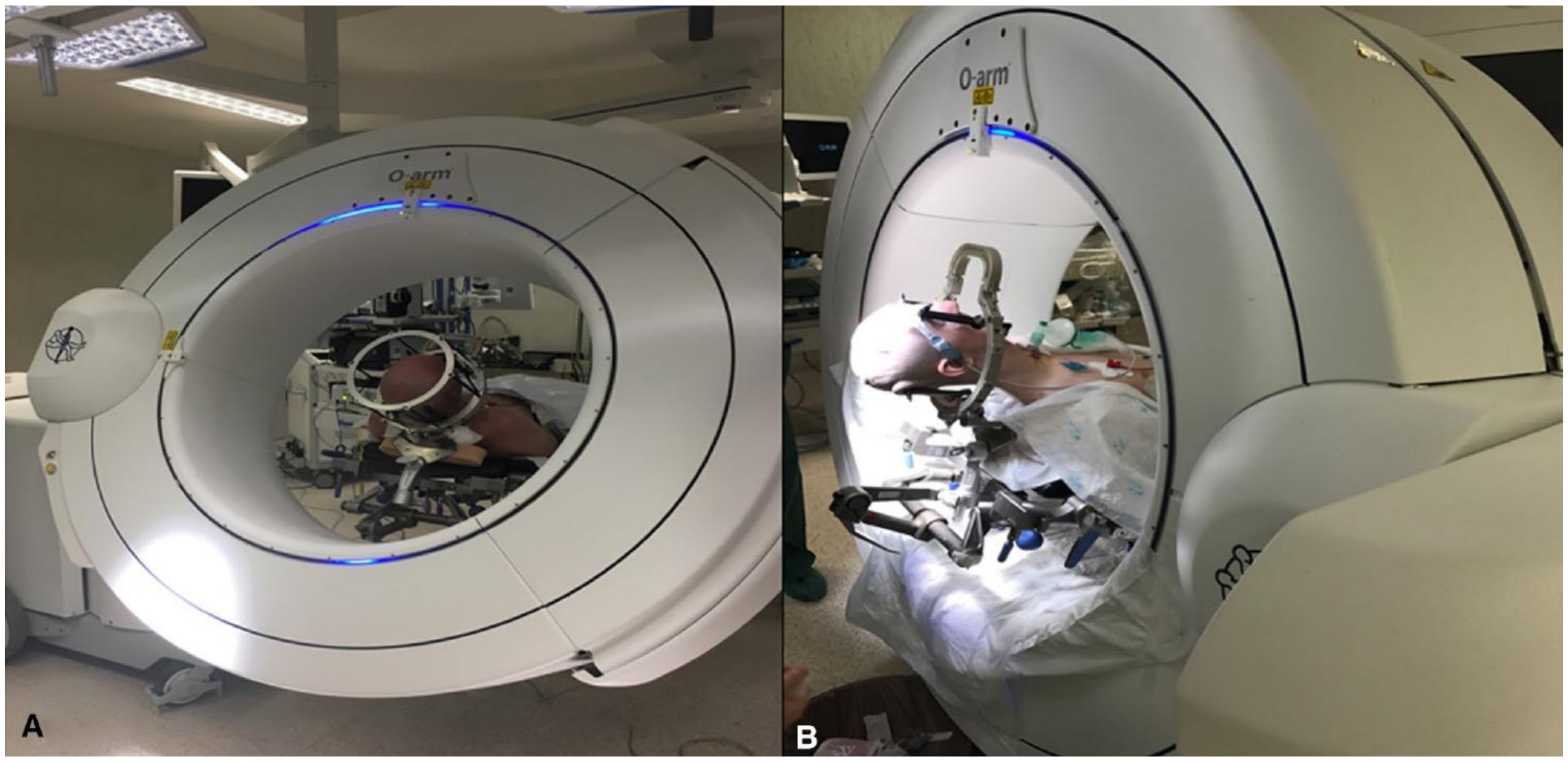

Supplementary Figure 1. O-arm imaging procedure. Figure 1A: the patient's head centered on the ring formed by the O-arm set for image acquisition (position 1). Figure 1B: the O-arm has been moved caudally to leave the surgical field free (position 2).

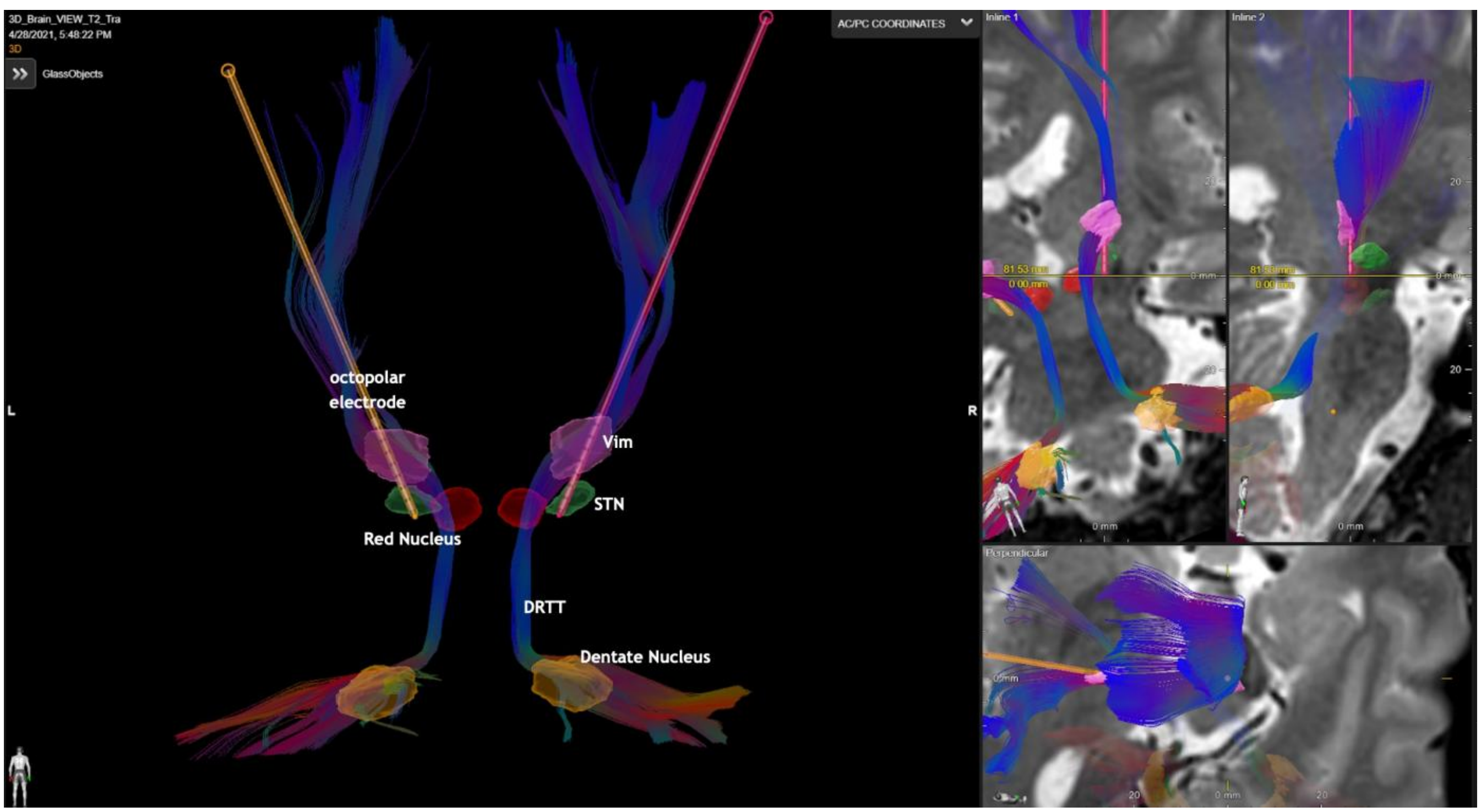

Supplementary Figure 2. Preoperative tractography image with direct DRTT reconstruction and representation of the octopolar electrode (Vercise™ Standard Lead DB2201-45-DC) and contacts location regarding VIM (proximal contacts) and PSA (distal contacts).

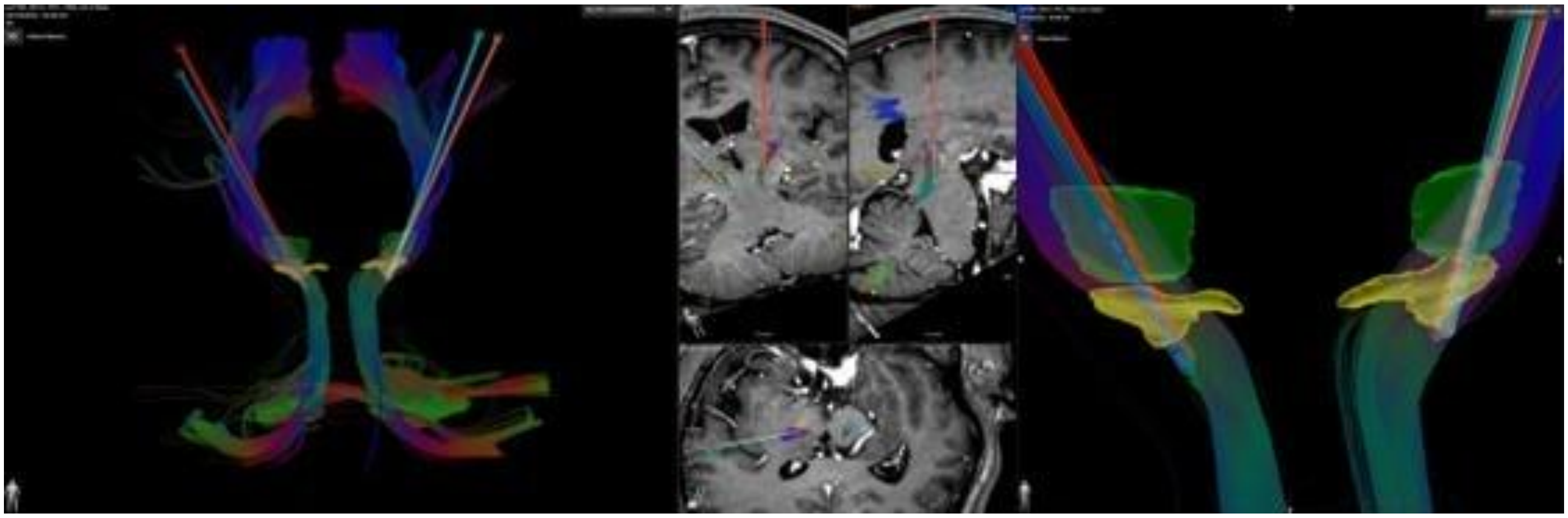

Supplementary Figure 3. Planned trajectories (red line) and final trajectories (blue line), with the position of the eight-contact electrodes in relation to the VIM model (green), the PSA/cZI (represented by cZI, in yellow), and the reconstructed DRTT, in one of the operated patients.
